# The Influence of Iron and Zinc Supplementation on Iron Apparent Absorption in Rats Fed Vitamins and Minerals Reduced Diets

**DOI:** 10.1007/s12011-020-02433-z

**Published:** 2020-10-18

**Authors:** Katarzyna Rolf, Olga Januszko, Joanna Frąckiewicz, Dawid Madej, Joanna Kaluza

**Affiliations:** 1 Department of Food Technology and Human Nutrition, University of Rzeszow, Rzeszow, Poland; 2 Department of Human Nutrition, Institute of Human Nutrition Sciences, Warsaw University of Life Sciences–SGGW, Warsaw, Poland

**Keywords:** Apparent absorption, Iron, Zinc, Supplementation, Rats, Reduced diets

## Abstract

Deficient human diet is usually reduced in many nutrients, but animal studies on iron absorption have been only carried out for rats fed well-balanced (control) and iron-deficient diets. The aim of this study was to evaluate the effect of iron or iron/zinc supplementation on iron apparent absorption (IAA) in rats fed a diet reduced in all vitamins and minerals (R). The study was conducted on 77, 6-week-old male Wistar rats in 3 stages as follows: stage I, 4-week period of adaptation to R diet (50% less vitamins and minerals compared to AIN-93M recommendations); stage II, 4-week supplementation period with iron (RSFe) or iron/zinc (RSFeZn); stage III, 2-week post-supplementation period (rats fed R diet). Feces samples to IAA determination were collected at the 20–22nd days of stage I and II and the 10–12th days of stage III. To determine the changes in IAA after introducing and discontinuation of supplementation, feces were collected for 3 days after introducing (stage II) and 5 days after the supplementation discontinuation (stage III). At the end of stage II, compared to R rats, the IAA was statistically significantly higher in RSFe and RSFeZn rats (30.3 ± 2.0% vs. 47.4 ± 1.2% and 51.0 ± 1.7%, respectively). After introducing iron or iron/zinc supplementation, the IAA stabilized fast already in the first day, while after discontinuation of the supplementation, at least 4 days was required to the stabilization. At the end of stage III, the IAA was significantly lower in RSFe (15.8 ± 6.6%) than in RSFeZn rats (43.4 ± 5.9%). In conclusion, to confirm that iron and zinc supplementation is more beneficial than iron supplementation only, especially after discontinuation of supplementation, further research among humans is necessary.

## Introduction

Iron and zinc are micronutrients necessary for the proper functioning of the organism. They perform catalytic, structural, and regulatory functions, e.g., they are a part of enzymes, participate in redox reactions, as well as they are necessary for a transport of oxygen and DNA synthesis [1, 2].

Anemia is one of the most common global diseases, occurs in a third of the world’s population, half of which is due to iron deficiency, most often among children and women, especially during pregnancy [3]. Anemia is also common in elderly, especially among vegetarians, people with celiac sprue, blood donators, people with inflammatory bowel disease (e.g., Crohn’s disease), and in those with chronic blood loss from the gastrointestinal tract [4, 5].

One of the most widely practiced public health strategy to prevent and treat iron deficiency anemia is iron supplementation, often used as multi-component dietary supplements [6–8].

Systematic review of trials showed 50% effectiveness of iron supplementation on reducing the risk of anemia and almost 80% on reducing risk of iron deficiency among children [9]. Introduction of iron supplementation could reduce global iron deficiency anemia by about 50% in pregnant and non-pregnant women [10].

Therefore, the World Health Organization (WHO) recommends interventional supplementation to women and adolescent girls living in settings where anemia is highly prevalent (≥ 40% of population) [11]. However, frequency of supplement intake depends on the country. In the USA, about 72% of pregnant women and 60% of lactating women used supplements with iron [12]. In Europe, in some countries (e.g., Norway, Poland), iron intake with usual diet in women in reproductive age is below recommendation, and about 23% of women took supplements containing iron. Simultaneously, it has been emphasized that supplementation should be individually recommended after evaluation of iron status [13, 14].

To prevent of iron deficiency, a mandatory fortification of some food products with iron can be also an effective strategy. For example, in the UK, to prevent iron deficiency, mandatory fortification of flour with iron was introduced [14]. Moreover, WHO recommends fortification of rice with iron, especially in settings where rice is a staple food [15].

On the other hand, excessive exposure to iron can lead to iron overload and increase risk of various diseases, including cardiovascular and cancer diseases in both humans [16] and animals [17]. Moreover, high doses of iron may interact with other diet ingredients including zinc, impact their absorption, excretion, and metabolism [1, 2]. It should be highlighted that iron supplementation should be carefully considered in areas where malaria is widespread because iron deficiency can have a protective effect against the development of this disease [4].

Although deficient human diet usually is reduced in many nutrients, animal studies on iron absorption have been carried out in rats fed well-balanced (control) and iron-deficient diets [18–21]. To the best of our knowledge, no studies have examined iron and zinc supplementation in relation to iron absorption in rats fed a diet reduced in many nutrients.

Therefore, the aim of this study was to evaluate the effect of iron or iron and zinc supplementation on iron apparent absorption in rats fed a diet reduced in vitamins and minerals. Diet used in the study, reduced in all vitamins and minerals, reflects common situation among adult people, when a diet is deficient in many nutrients and only one or two micronutrients are supplemented in high doses [10, 11, 22]. The presented study is a continuation of our previously published results, where rats were fed control and iron-deficient diet only [21].

## Materials and Methods

### Animals and Diets

The study was conducted on 77 certified 6-week-old male Wistar rats, with a mean initial weight of 296 ± 23 g. Animals were obtained from the Medical Research Centre of Polish Academy of Sciences in Warsaw (NIHCertified, No. A5438-01). All rats were housed individually in glass propylene cages. Living conditions were controlled as follows: temperature 21–22 °C, humidity 50–60%, and 12 h light cycle. Rats had access to water ad libitum, and the amount of forage consumed by every rat was recorded on a daily basis. The study was performed according to the guidelines and approval of the Third Local Ethics Commission for Experiments on Animals in Warsaw.

Depending on a group and a stage of the experiment, rats were fed three types of diets. The reduced diet (R) contained 50% less all vitamins and minerals compared to the AIN-93M recommendations [23]. The diet supplemented with iron (RSFe) contained about 10 times higher amount of iron compared to the AIN-93M recommendations, while the diet supplemented with iron and zinc (RSFeZn) contained about 10 times higher of iron and zinc compared the AIN-93M recommendations [23]. A 1 kg of diet included wheat starch (643 g), casein (140 g), sucrose (100 g), cellulose (50 g), soybean oil (40 g), choline bitartrate (2.5 g), L-cysteine (1.8 g), t-buthylhydroquinone (0.008 g), mineral mixture (17.5 g), vitamin mixture AIN-93-VX of MP Biomedicals, LLC company, Cat. No. 960402 (5 g). Iron and zinc were added to all diets in ferric citrate and zinc carbonate form. Levels of these minerals in diets were determined by flame atomic absorption spectrometry (FAAS), using a UNICAM 989, SOLLAR (UK) spectrophotometer, and were presented previously (Table 1) [24].

**Table 1:** Content of iron and zinc level in experimental diets [24]

Content*	Diet/group		
	R	RSFe	RSFeZn
Iron (mg/1 kg dry diet)	32.3	501	542
Zinc (mg/1 kg dry diet)	28.2	29	500

*R*, reduced diet in all vitamins and minerals; *RSFe*, reduced diet supplemented with iron; *RSFeZn*, reduced diet supplemented with iron and zinc

*Moreover diets included (per kg diet) wheat starch 643 g, casein 140 g, sucrose 100 g, soybean oil 40 g, cellulose 50 g, modified mineral mix AIN-93 M 17.5 g, vitamin mix AIN-93-VX (MP Biomedicals, LLC, No. 960402) 5 g, L-cysteine 1.8 g, choline bitartrate 2.5 g, and t-bithlyhydroquinone 0.008 g

^#^The contents of iron and zinc in diets were determined by FAAS and were presented as means of 6 diet samples (each determined in triplicate)

### Design of the Study

The study design is presented in Table 2. Before starting of the experiment, all animals were fed the AIN-93M diet [23] for 14 days. After rats’ adaptation to laboratory conditions, rats were divided into groups, of 6–7 individuals each, and named R, RSFe, and RSFeZn. Rats to the particular groups were selected based on their initial body weight in order to the averages of body weight between groups did not differ statistically significant. In stage I, all rats were fed the R diet by 4 weeks. In stage II, chosen groups of rats were fed the R diet supplemented with iron (RSFe group) or iron and zinc (RSFeZn group). After 4-week of supplementation stage, in stage III, the rats were fed the R diet by 2 weeks.

**Table 2:** Design of the study

Group	Stage I, adaptation to diets (4 weeks)	Stage II, supplementation period (4 weeks)	Stage III, post-supplementation period (2 weeks)
R	R diet	R diet	R diet
RSFe		RSFe diet	
RSFeZn		RSFeZn diet	

*R*, reduced diet in all vitamins and minerals; *RSFe*, reduced diet supplemented with iron; *RSFeZn*, reduced diet supplemented with iron and zinc. Number of animals is 6–7 in each group

The amount of feed intake by the rats and feed spills were recorded everyday separately for each animal, while the rats’ body weight was recorded weekly. Based on the weekly feed intake and weight gain of rats, a feed conversion ratio (FCR) was calculated as feed intake (g) to weight gain of rats (g) per week.

### Blood Collection and Hematological Parameter Determination

After each stage of the experiment and overnight fasting, the rats were anesthetized by thiopental intraperitoneal injection. Blood was collected by puncture of the heart and transferred into tubes containing potassium EDTA. On the day of blood collection, without prior freezing, hematological parameters such as red blood cell (RBC) count, hemoglobin (HB), hematocrit (HTC), and mean corpuscular volume (MCV) were determined in the Laboratory of the Veterinary Center of Warsaw University of Life Sciences (Warsaw, Poland) with an Abacus Junior Vet Analyzer (Diatron MI PLC, USA).

### Iron Apparent Absorption

In order to determine the iron apparent absorption (IAA), feces were collected from randomly selected animals (6–7 from each group) for 3 consecutive days between 20th and 22nd day of stage I and stage II, and on days 10–12th of stage III. To determine the changes in the IAA after introducing and discontinuation of supplementation, feces were collected for the first 3 days after introducing (stage II) and for the first 5 days after discontinuation of the supplementation (stage III). All collected samples were dried at 105 °C for 3 h, cleaned of food residue, re-dried, and then mineralized for 20 min in 65% HNO3 (no. 1.00456, Merck, Darmstadt, Germany) in a microwave digestion system (Mars5, CEM, Matthews, USA) at the temperature of 210 °C and pressure 210 PSI.

Levels of iron and zinc in samples were determined by flame atomic absorption spectrometry (FAAS) using UNICAM 989 SOLLAR (UK) spectrophotometer. Standard curves for determining of iron and zinc were prepared by diluting iron (Merck 1.19781) and zinc (Merck 1.19806) standard reference materials in a range from 0 to 5.0 μg/cm^3^. To test the method accuracy, the serum certified reference material (Seronorm™ Trace Elements Serum L-1, JL4409) was used. The iron and zinc recovery for serum was 102.0% and 99.9%, respectively, and the coefficients of variation were 2.6% for iron and 6.3% for zinc.

The IAA was calculated using the following formula

$$ \mathrm{IAA}=\left[\left(I\hbox{--} E\right)/\mathrm{I}\right]\times 100 $$

where IAA is the iron apparent absorption (%), *I* is the daily intake of iron (mg), *E* is the daily excretion of iron with feces (mg).

### Statistical Analysis

Data were analyzed using the Statistica software version 13PL. The results were presented as the mean ± standard error (SE). The effects of supplementation were evaluated based on Kruskal-Wallis test; comparisons between groups were conducted using Mann-Whitney *U* test. The results with *p* values ≤ 0.05 were considered as statistically significant.

## Results

### Body Weight, Feed, Iron and Zinc Intake

Initial and final body weight, as well as weekly weight gain, did not differ between rats fed various type of R diets within each stage of the experiment (Table 3). Moreover, within each stage of the study, amounts of feed intake and FCR also did not differ between experimental groups. Expectedly, in the supplementation period (stage II), intake of iron was significantly higher in RSFe and RSFeZn rats compared to R rats, and zinc intake was significantly higher in RSFeZn rats compared to R and RSFe rats.

**Table 3:** Effect of iron or iron and zinc supplementation on the body weight, feed, iron and zinc intake after adaptation to diets (stage I), supplementation (stage II), and post-supplementation (stage III) periods (mean ± SE)

Group	Initial body weight (g)	Final body weight (g)	Weight gain (g) per week	Feed intake (g) per 100 g body weight	FCR*	Daily iron intake (mg) per 100 g body weight	Daily zinc intake (mg) per 100 g body weight
Stage I							
R	295 ± 7.3	352 ± 6.5	14.1 ± 1.6	6.7 ± 0.2	3.5 ± 0.4	0.24 ± 0.01	0.24 ± 0.01
Stage II							
R	343 ± 9.9	382 ± 9.2	9.4 ± 0.6	6.8 ± 0.2	4.7 ± 1.3	0.21 ± 0.01 ^a^	0.20 ± 0.01 ^a^
RSFe	351 ± 10.9	385 ± 13.9	8.5 ± 1.0	6.6 ± 0.2	5.1 ± 0.9	3.28 ± 0.11 ^b^	0.19 ± 0.01 ^a^
RSFeZn	344 ± 8.1	378 ± 9.5	8.5 ± 0.7	6.9 ± 0.2	5.0 ± 0.5	3.43 ± 0.09 ^b^	3.42 ± 0.08 ^b^
Stage III							
R	387 ± 10.2	402 ± 7.8	3.9 ± 0.1	7.1 ± 0.2	23.2 ± 0.1	0.21 ± 0.01	0.22 ± 0.01
RSFe	385 ± 8.8	398 ± 8.5	3.6 ± 0.1	7.0 ± 0.2	23.1 ± 0.3	0.21 ± 0.02	0.19 ± 0.01
RSFeZn	382 ± 9.1	395 ± 9.2	3.7 ± 0.1	7.3 ± 0.2	23.5 ± 0.2	0.22 ± 0.01	0.19 ± 0.02

*R*, reduced diet in all vitamins and minerals; *RSFe*, reduced diet supplemented with iron; *RSFeZn*, reduced diet supplemented with iron and zinc

^a, b^Different letters indicate a statistically significant differences within a stage of the experiment (*p* value ≤ 0.05, Mann-Whitney *U* test)

*FCR, feed conversion ratio was calculated as feed intake (g) to weight gain of rats (g) per week

Number of animals is 6–7 in each group

### Hematology Parameters, Iron and Zinc Serum, and Liver Concentration

Hematology parameters did not differ between rats fed the various type of R diets within each stage of the experiment (Table 4). However, statistically significant increase RBC, HB, and HTC and decrease MCV were observed in RSFeZn group after the post-supplementation period (stage III) compared to these rats after the intervention period (stage II). For RSFe, group similar associations were found, but only for RBC, the significant difference was found.

**Table 4:** Effect of iron or iron and zinc supplementation on hematology parameters and total iron binding capacity (TIBC) after adaptation to diets (stage I), supplementation (stage II), and post-supplementation (stage III) periods (mean ± SE)

Group	RBC (10^6^/μL)	HB (g/dL)	HTC (%)	MCV (fl)	TIBC (μg/dL)
Stage I					
R	8.6 ± 0.2	14.7 ± 0.3	41.1 ± 1.1	48.0 ± 0.3	441 ± 21
Stage II					
R	8.9 ± 0.3	14.6 ± 0.5	42.8 ± 1.3	48.2 ± 0.5*	510 ± 24
RSFe	8.4 ± 0.4*	14.2 ± 0.4	40.4 ± 2.0	47.9 ± 0.5	456 ± 21
RSFeZn	8.1 ± 0.2*	13.7 ± 0.2*	39.1 ± 0.8*	48.1 ± 0.4*	444 ± 30
Stage III					
R	9.0 ± 0.5	14.0 ± 0.6	43.5 ± 1.0	46.6 ± 0.5	498 ± 16
RSFe	9.5 ± 0.2	14.9 ± 0.3	44.4 ± 1.1	46.4 ± 0.7	428 ± 17
RSFeZn	9.3 ± 0.1	14.2 ± 0.2	43.0 ± 0.5	46.0 ± 0.6	486 ± 26

*HB*, hemoglobin; *MCV*, mean corpuscular volume; *HTC*, hematocrit; *RBC*, red blood cells; *R*, reduced diet in all vitamins and minerals; *RSFe*, reduced diet supplemented with iron; *RSFeZn*, reduced diet supplemented with iron and zinc; *TIBC*, total iron binding capacity

*A statistically significant difference between stage II and stage III (*p* value ≤ 0.05, Mann-Whitney *U* test)

Number of animals is 5–7 in each group

After supplementation (stage II) and post-supplementation (stage III) periods, iron levels in the liver were significantly higher in RSFe and RSFeZn compared to R rats, while zinc levels were significantly higher in RSFeZn (but not RSFe) compared to R rats only after stage II. An effect of the intervention on serum concentration of iron and zinc in rats fed the various types of R diets within each stage of the experiment was not found. Detailed results on iron and zinc concentration in serum and the rats’ organs were published previously [24].

### The IAA at the End of Experimental Stages

**Fig. 1:**
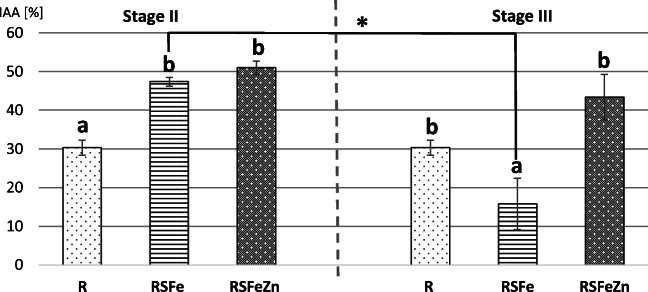
Average of the iron apparent absorption (IAA) in rats fed different types of diets in the study. Stage II, supplementation period; stage III, post-supplementation period; R, reduced diet, feces samples collected in 20–22nd days; RSFe (stage II), reduced diet supplemented with iron, feces samples collected in 20–22nd days; RSFeZn (stage II), reduced diet supplemented with iron and zinc, feces samples collected in 20–22nd days; RSFe (stage III), feces samples collected in 10–12th days; RSFeZn (stage III), feces samples collected in 10–12th days. (a, b) Different letters indicate a statistically significant difference within a stage of the experiment; Asterisk indicates a statistically significant difference between stage II and stage III (*p* value ≤ 0.05, Mann-Whitney *U* test). Bars represented mean ± SE of 6–7 animals

In the end of stage I (the 20–22nd days), the IAA was 44.09 ± 3.5% in R rats, and in the end of stage II (the 20–22nd days), a statistically significant decrease in the IAA, to the value 30.38 ± 2.0%, was observed. In the end of supplementation period (stage II), the IAA in rats fed RSFe diet (47.41 ± 1.2%) and rats fed RSFeZn diet (50.96 ± 1.7%) was statistically significantly higher compared to rats fed R diet (Fig. 1). In the end of post-supplementation period (stage III), the average of IAA in the RSFe group but not in RSFeZN group decreased significantly compared to stage II, and was 15.76 ± 6.6% in RSFe rats and 43.43 ± 5.9% in RSFeZn rats. Moreover, in the end of stage III, the IAA in RSFe rats was significantly lower in comparison to R and RSFeZn rats.

### Changes in the IAA in Consecutive Days After Supplementation Introducing and Discontinuation

**Fig. 2:**
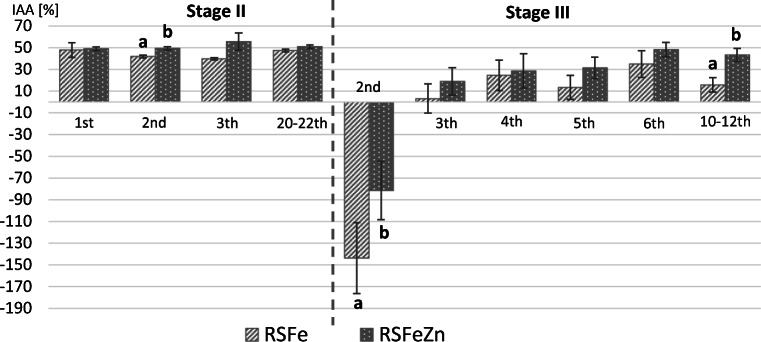
The iron apparent absorption (IAA) during consecutive days after introducing and discontinuation of iron and zinc supplementation. Stage II, supplementation period; stage III, post-supplementation period; RSFe, reduced diet supplemented with iron; RSFeZn, reduced diet supplemented with iron and zinc. (a, b) Different letters indicate a statistically significant difference within a stage of the experiment (*p* value ≤ 0.05, Mann-Whitney *U* test). Bars represented mean ± SE of 6–7 animals

The IAA during consecutive days after introducing of iron or iron and zinc supplementation is presented in Fig. 2. In the first days after introducing iron as well as iron and zinc supplementation (stage II), the IAA did not change substantially compared to R group (the end of stage I, 44.09 ± 3.5%). Although, in the second day after introducing of the supplementation, the IAA was statistically significantly lower in RSFe rats (41.92 ± 1.3%) compared to RSFeZn rats (49.39 ± 1.7%), at the end of stage II (days 20–22nd) did not differ significantly between both groups of rats, and stabilized at level 47.41 ± 1.2% in RSFe rats and 50.96 ± 1.7% in RSFeZn rats.

Discontinuation of the supplementation (stage III) resulted high excretion of iron already in the second day in both groups of rats (Fig. 2). The values of IAA were negative in RSFe group (IAA = − 143.76 ± 32.7%) and in RSFeZn group (IAA = − 81.40 ± 26.9%), and differed statistically significant between these groups. In the third day of stage III, the IAA reached positive values and stabilized between the 10 and 12th days at the level 15.76 ± 6.6% in RSFe group and 43.43 ± 5.9% in RSFeZn group. In the consecutive days after discontinuation of the supplementation, the IAA in rats fed RSFeZn diet was higher than in rats fed RSFe diet; however, the statistically significant differences between the groups were found only in the 2nd and the 10–12th days. Based on this part of the study, we may conclude that after discontinuation of the supplementation, at least 4 days was required to IAA stabilization.

## Discussion

In the presented study, at the end of the supplementation period, the IAA did not differ between RSFe and RSFeZn rats and was stabilized at the level of 47.41% in RSFe and 50.96% in RSFeZn rats. This result may indicate that zinc administered simultaneously with iron dose did not disturb IAA in rats fed a diet reduced in all vitamins and minerals. Different results were obtained in our previous study in rats fed iron-deficient diet only; iron absorption in a group supplemented with iron only was statistically significantly higher than in rats supplemented with iron and zinc (57.4 ± 2.3% vs. 49.4 ± 2.1%, respectively) [21]. It should be pointed that in this study, as well as previous study [21], the level of IAA was not dependent on body weight and amounts of feed intake by rats within each stage of the experiment. In other experiment, in which animals received a double amount of iron compared with the presented study, IAA was almost the same as in our study and oscillated at 50% in both iron as well as iron and zinc supplemented groups [19]. In contrast, in a study where rats were fed a well-balanced diet supplemented with iron (iron amount 3 times higher than in RSFe diet of our experiment), an average value of IAA at the end of the stage was negative (− 4.5%); however, in a group supplemented with zinc only, a level of IAA was similar to RSFeZn rats of our study (46.8%). In that study, there was not a group of rats fed a diet with combined iron and zinc supplementation [18]. These results indicate that supplementation of well-balanced diet should not be recommended and iron absorption depends not only on its amount in the diet but also on other diet ingredients. For example, the compounds contained in the diet, such as organic acids (e.g., lactic and ascorbic), as well as fructose, sorbitol, and amino acids (like cysteine or lysine), enhance non-hem iron absorption. On the other hand, phytates, phosphates, tannins, calcium, and some other food compounds may make the absorption more difficult [5, 25].

A level of IAA may depend on the chemical form of iron and zinc used in an experimental diet. Akhtar et al. [20] obtained the highest IAA in rats for NaFeEDTA (57%) and the worst result for elemental iron (37%), lower even than for control diet (43%). In Januszko et al.’s [21] study and in our present study, iron citrate and zinc carbonate were used in rats’ diets, which make comparison easier between both studies. Both forms are strictly defined to use in research with animal rodents by the AIN-93 diet [23] as well as they are authorized for supplementation by Commission Regulation (EC) No 1170/2009 in dietary supplements intended for humans. The highest average relative bioavailability of iron is provided by water-soluble compounds, but those water-insoluble could also be well absorbed but more slowly. Ferrous sulfate and lactate, as well as ferric ammonium citrate and sulfate, are well absorbed by rats, and ferrous sulfate, lactate, and fumarate in human [26]. In a study on human epithelial adenocarcinoma, ferrous sulfate had significantly higher absorption compared with ferrous fumarate and gluconate [27]. WHO suggested forms of iron for human supplementation are ferrous sulfate, fumarate, and gluconate [11]. They are the most often used forms of iron in supplements [27]. However, in a meta-analysis, it has been shown that ferrous sulfate increases the risk of gastrointestinal-specific side-effects among adults [28]. In another meta-analysis, it has been found that overall and gastrointestinal adverse events were among 32% of participants who used ferrous sulfate, among 47% of those who used ferrous fumarate and among 31% of people with ferrous gluconate supplementation [29].

In this study, discontinuation of supplementation was associated with a rapid decrease of iron absorption to negative values in the second day of stage III. Similar results were obtained in our previous study [21]. In that study, IAA decreased to negative values on the second day after discontinuation of iron but not iron and zinc supplementation. It should be highlighted that in our study, IAA stabilized in the 10–12 days after RSFe supplementation at a level of 15.76% and this level was significantly lower than those obtained after RSFeZn supplementation (43.43%). In the study of Januszko et al. [21] at the end of the post-supplementation period, IAA increased to 56.4% in rats fed iron and to 56.6% in rats fed iron and zinc supplemented diet. The difference may depend on diet use in both studies, i.e., iron deficiency diet in Januszko et al. [21] study and diet reduced in all vitamins and minerals in our work. This result indicates that combined iron and zinc supplementation was more efficient than iron supplementation only in rats fed a diet with reduced amounts of all vitamins and minerals. In the case of a well-balanced diet, no additional benefits of zinc supplementation were observed for iron absorption.

According to study results, the combined iron and zinc supplementation is effective in the prevention and treatment of anemia in humans and positively affects iron parameters in the blood [30, 31], especially ferritin [32]. Combined iron and zinc supplementation compared to supplementation with iron or zinc alone (especially in long-term use) also seems to be more effective on psychomotor development and orientation of infants [33]. Nevertheless, the efficiency of the supplementation may depend on the dose of iron and zinc. It is known that the excess of zinc may inhibit iron absorption and vice versa [34]. It should also be pointed that iron, as well as zinc, affects the metabolism of other ingredients, e.g., vitamin A. In malnourished children with anemia, iron and zinc supplementation improved the parameters of vitamin A in the blood (i.e., plasma retinol) although supplementation of iron alone was slightly more beneficial [32].

Several mechanisms may explain the beneficial effect of zinc supplementation on iron absorption. Divalent metal transporter 1 (DMT1) transports iron in enterocytes. Zinc is a ligand for DMT1; however, its transport is limited in relation to iron. Moreover, zinc induces ferroportin transcription, transmembrane iron transporter, and it stabilizes a level of duodenal cytochrome b, which plays a crucial role in dietary iron absorption. The presented mechanisms can be disrupted by high iron as well as high zinc intake [35]. In our study, in rats fed a diet reduced in all vitamins and minerals, an adverse effect of zinc together with iron supplementation on iron apparent absorption was not observed. We hypothesized that in the situation of moderate deficiency in the diet of many nutrients, zinc supplemented together with iron did not impair iron absorption by its participating in the above mechanisms involved in iron status regulation. In our experiment, the simultaneous iron and zinc supplementation did not affect adversely hematology parameters and iron concentration in serum and the liver, which also may prove that this supplementation had no adverse effect on iron apparent absorption.

The relevance of our results included establishment whether in rats with moderate deficiency vitamins and minerals in a diet simultaneous zinc supplementation together with an iron supplement will be disturb iron absorption. Feeding rats the diet reduced in all vitamins and minerals and testing the effect of discontinuation of the supplementation may reflect common situation among humans when a diet is deficient in many nutrients, but only one is used as a supplement in high doses, and when dietary correction is not made after cessation of the supplementation. The novelty of the study was introducing the post-supplementation observation, which allows us to determine that after discontinuation of the supplementation stabilization of iron apparent absorption involved more time. Another strength of this study is the use of the controlled laboratory conditions in our experiment which allowed us to eliminate confounding factors; however, this is also a limitation of the study, due to inability of direct transfer of results to human. The next limitation of our experiment is that rats were supplemented using only one specified chemical form of iron and zinc (i.e., ferric citrate and zinc carbonate, respectively). Using other chemical forms of minerals, obtained results may differ. Moreover, the study was conducted on 6-week-old male Wistar rats. Presumably that using in experiment animals in different ages, other strain or female may influence results as well.

## Conclusions

Results of our study indicate that combined iron and zinc supplementation, compared to iron supplementation alone, may be more effective in iron apparent absorption in the situation of all vitamins and minerals deficiency in a diet. In rats fed a diet with reduced amounts of vitamins and minerals, iron apparent absorption stabilizes fast after introducing iron or iron and zinc supplementation (already in the first day), while after discontinuation of the supplementation to stabilization, more time was required (at least 4 days). To confirm whether zinc addition to iron supplementation is more beneficial than iron supplementation only, further research among humans is necessary.
